# Perceived Barriers to Micronutrient-Rich Diets Among Charitable-Service Users in Two European Settings: A Qualitative Pilot Study

**DOI:** 10.3390/foods15193515

**Published:** 2026-10-01

**Authors:** Aikaterini Palascha, Aneta Janikowska, Amber Peeters, Betty Chang, Siân Astley

**Affiliations:** 1 European Food Information Council (EUFIC AISBL), 1040 Brussels, Belgium; amber.peeters@eufic.org (A.P.); betty.chang@eufic.org (B.C.); 2 Food Bank in Olsztyn, 10-166 Olsztyn, Poland; aneta.janikowska@bankizywnosci.pl; 3 European Food Information Resource (EuroFIR AISBL), 1050 Brussels, Belgium; sa@eurofir.org

**Keywords:** micronutrient(s) deficiency/ies, AAAQ framework, nutritional inequalities, socioeconomic disadvantage, food assistance, refugees, qualitative research, Zero Hidden Hunger EU, hidden hunger

## Abstract

People experiencing socioeconomic disadvantage are disproportionately affected by micronutrient deficiencies, but evidence concerning the barriers to micronutrient-rich diets among disadvantaged populations in European settings is limited. This qualitative study (eight focus groups) explored the experiences of 68 beneficiaries of charitable organisations in Trieste, Italy (*n* = 26), and Olsztyn, Poland (*n* = 42) (i.e., food-assistance recipients and refugees, including asylum seekers). Data were analysed using thematic analysis informed by an adapted Availability, Accessibility, Acceptability, and Quality (AAAQ) framework. Participants described intersecting economic, physical, temporal, informational, social, cultural, and organisational barriers to accessing and consuming micronutrient-rich foods. High food prices and competing household expenses constrained food choice and variety, while transport, work schedules, cooking and storage restrictions, culturally incompatible provision, social influences, and limited access to professional guidance created additional barriers. Availability and perceived quality differed between retail, food-assistance, and accommodation settings. Participants generally preferred obtaining micronutrients through food, expressed predominantly negative attitudes towards fortified foods, and reported divergent attitudes towards food supplements. The study did not assess dietary intake or micronutrient status but identified perceived barriers that might increase vulnerability to inadequate intake. The findings support context-sensitive, multilevel responses spanning food assistance, social protection, public health, organisational provision, and nutrition communication.

## 1. Introduction

Often undetected, micronutrient deficiencies (i.e., deficient levels of one or more vitamins or minerals in the body) can have lasting health implications due to the role of micronutrients in growth, immunity, metabolic functions, and reduced risk of chronic diseases including cardiovascular diseases and cancer [1]. Micronutrient deficiencies affect significant numbers of the adult population in Europe, with the burden falling disproportionately on older adults [2], females [3], people living in central and eastern European countries [3,4], and people with socioeconomic disadvantage [5], highlighting clear health inequalities.

Socioeconomic status is a key determinant of dietary quality and micronutrient intake [6,7]. Those in lower socioeconomic strata more frequently have poorer quality diets [8], resulting from intersecting economic, contextual, and (inter)personal factors [9,10,11]. Consistent with this, food insecurity is also associated with micronutrient deficiencies such as iron and vitamin A [12]. Vulnerability to poor diets and micronutrient deficiencies is further exacerbated among ethnic minorities, immigrants, and refugees; groups that often also face socioeconomic disadvantage [13,14]. For example, in a systematic review of micronutrient adequacy (i.e., adequacy of micronutrient intake relative to dietary recommendations), Ngo et al. [15] found that deficiencies in folate, vitamin B12, calcium, and iron affected over half of adult minority populations in Europe (i.e., migrants from ethnic groups outside the former EU-15). In addition, the prevalence of vitamin D [16,17] and iron [18,19,20] deficiencies among immigrants in European countries (e.g., Italy, Finland, the Netherlands), is greater than in non-migrant populations. Given that Europe is a region with significant numbers of displaced individuals globally [21], these patterns raise particular concern because micronutrient deficiencies impact not only individual health but also productivity and national economies [22].

Despite this body of evidence, important knowledge gaps remain regarding factors driving consumption of diets poor in micronutrients and increased vulnerability to micronutrient deficiencies among socioeconomically disadvantaged groups. A qualitative study by Stavitz [23] found that personal dietary habits (e.g., preference for unhealthy food), social influences (e.g., peer pressure to eat unhealthily), community resources (e.g., limited accessibility of food stores), and broader socioeconomic factors (e.g., rising food prices, economic disparities) collectively limit micronutrient access. However, the study included a diverse group of participants with varied socioeconomic status and did not specify their geographical location, limiting contextual interpretation. Likewise, a study in Poland with adults across diverse sexes, ages, education levels, and body weight statuses observed limited knowledge about vitamin D and calcium, in particular their recommended intakes, interactions with other nutrients, as well as risk factors and consequences of deficiencies [24], potentially hindering adequate vitamin D intake. Studies that have investigated barriers to healthy eating among low income groups mainly cite factors related to affordability and accessibility [25], although food preferences and perceptions of nutrition information being personally irrelevant and/or contradictory have also been reported [10]. Similar barriers have been identified among refugees and immigrants in high-income countries, along with the limited availability of traditional foods and lack of social support, which constitute additional barriers unique to these groups [26]. Notably, most studies addressing these factors have either been conducted outside Europe, relied on socioeconomically heterogeneous samples, or not focus specifically on micronutrient adequacy. As a result, understanding of specific structural, sociocultural, and institutional contexts that shape vulnerability to micronutrient deficiencies among the most disadvantaged groups within European settings are limited. Moreover, the perspectives of marginalised groups, such as refugees or asylum seekers, have not received enough attention, despite the additional hardships these groups often encounter (e.g., displacement, cultural and linguistic constraints, resource scarcity and food insecurity) [14].

To address these gaps, this qualitative pilot study used focus-group discussions with charitable food-assistance beneficiaries and refugees, including asylum seekers, recruited through charitable organisations in Trieste (Italy) and Olsztyn (Poland) that had pre-established relationships with the project partners and prior experience participating in similar research projects. It explored participants’ perceptions and experiences of factors that might limit access to, and consumption of, micronutrient-rich foods. It was hypothesized that participants experience multiple and interconnected barriers that limit their access to and consumption of micronutrient-rich foods, including economic constraints, limited food availability, accessibility and acceptability, sociocultural factors, as well as knowledge gaps and misconceptions. Refugees and asylum seekers were further expected to encounter additional barriers compared to native participants.

This research was informed by an adapted Availability, Accessibility, Acceptability, and Quality (AAAQ) framework, originally developed in relation to the right to health [27]. The framework was used to organise participants’ accounts of availability and quality of micronutrient-rich foods, their physical, temporal, and economic accessibility, and their personal and sociocultural acceptability, alongside the accessibility of information in this area. By identifying the multiple barriers across the AAAQ dimensions that may constrain access to and consumption of micronutrient-rich foods among socioeconomically disadvantaged populations this research highlights that access to sufficient food does not necessarily translate into access to a nutritionally adequate diet. This distinction is particularly relevant to addressing hidden hunger, characterized by inadequate intake of micronutrients despite adequate energy intake, and underscores the need to move beyond a food-sufficiency model focused primarily on the quantity of food available and the prevention of hunger towards a nutrient-sufficiency model that prioritizes nutritional adequacy and diet quality. The findings are particularly relevant to charitable food providers, non-governmental organisations, public health practitioners, policymakers and researchers working with comparable populations and service settings.

## 2. Materials and Methods

### 2.1. Participant Recruitment

Two charitable organisations, Caritas Trieste (Italy) and Food Bank Olsztyn (Poland), facilitated access to the target populations. Staff used purposive sampling to recruit: (a) beneficiaries of their food assistance programmes (i.e., social supermarket in Trieste and the food bank in Olsztyn), and (b) refugees, including asylum seekers, who were accessing other services provided by the organisations (e.g., language courses, psychological support, legal counselling, job placements). Participants were grouped according to the service through which they had been recruited to allow their experiences to be discussed in greater detail. Individuals who were 18 years old or older and able to provide informed consent were eligible. Eligibility for the food-assistance programmes was determined by the respective organisations using financial, social and situational criteria, including income, unemployment, disability and illness.

Social workers and volunteers affiliated with the organisations approached eligible individuals in person (e.g., during shopping visits at the social supermarket, upon delivery of parcels of the food bank, during the delivery of other services) to explain the study briefly as well as their rights as participants (i.e., informed consent, right to withdraw, assertion that participation did not affect access to services), and provided them with a leaflet for further information. Recruiters encouraged individuals to contact the organisation if they had questions or wanted to participate. Each organisation conducted four focus groups, comprising two with food-assistance beneficiaries and two with refugees, to provide more comprehensive understanding of the issues per stratum, as recommended by Hennink et al. [28]. The aim was to invite 12–15 participants per focus group, anticipating non-attendance and seeking a minimum attendance of six participants, in line with previous literature [29].

### 2.2. Data Collection and Material

FGs took place at the premises of the organisations between June and July 2026. Upon arrival, participants were welcomed and asked to sign the informed consent form. Participants completed a questionnaire collecting sociodemographic information (see Table S1 in Supplementary Materials). Discussions were facilitated by leadership-level staff from the organisations (male facilitator in Italy, female in Poland); both had prior experience with conducting FGs. Prior to the study, facilitators participated in an one-hour training session offered by the researchers. This training provided practical guidance on recruiting participants, setting up the focus groups, ensuring participant anonymity, using the discussion guide, and facilitating discussions in a manner minimising biases (e.g., neutral probing, encouraging equal participation). Observers (i.e., other staff of the organisations), who were present during the discussions, took field notes about key points discussed, non-verbal communication (e.g., emotions, changes in tone, use of sarcasm or humour), and the focus group context (e.g., reasons for late arrival). Field notes were used to support the analysis by providing context for participant responses.

Facilitators explained the purpose of the study and the procedures for conducting and recording the discussions. They explained the rights of participants and encouraged them to express their ideas openly. After a few initial questions assessing knowledge and perceptions about roles and sources of micronutrients, facilitators presented an infographic about the food sources richest in vitamins and minerals (see Supplementary Materials). The infographic was intended to establish common terminology and a shared basis for the subsequent discussion. Throughout the discussion, the term “nutrient-rich foods” was used to refer to foods that are good sources of vitamins and/or minerals (i.e., fruits, vegetables, pulses, wholegrains, fish/seafood, dairy, meat, eggs, nuts/seeds). The infographic remained visible during discussions. The discussion guide included 31 questions and follow up enquiries covering: knowledge about micronutrients, experiences with receiving advice on preventing deficiencies, perceived dietary variety (as a distal proxy of micronutrient adequacy) and factors impacting it, perceived barriers associated with the availability, affordability, accessibility, acceptability, and quality of micronutrient-rich foods (in and out of the context of food assistance), impact of migration (for refugees), social environment, culture/religion, work environment, mental health, living conditions, and food storage/preparation practices on access to and consumption of micronutrient-rich foods, perceptions about fortified foods, sun exposure, and food supplements, and information needs about micronutrients. Although the discussion guide was not formally pilot-tested prior to data collection, the questions were reviewed by the research team and the facilitators to ensure their relevance, clarity, and appropriateness for addressing the study aims. The discussion guide was flexible and facilitators were allowed to skip questions if they had already been addressed by the preceding discussion. At the end, facilitators wrapped up key takeaways and encouraged participants to share any last thoughts.

FGs lasted between 38 and 111 min (M = 85 min, Mdn = 95 min). The two FGs with refugees in Italy, were shorter than the rest (38 and 48 min, respectively) due to a public transportation strike and linguistic challenges that limited exchanges. Nevertheless, all topics were brought up even in shorter FGs. The FGs were conducted in local languages, i.e., Italian and Polish; interpreters and language support were not available. Those not fluent in Italian or Polish were unable to provide informed consent and therefore excluded. All materials were available in both local languages and English (see Supplementary Material for English versions). Discussions were recorded (audio only), transcribed verbatim in Polish or Italian and then translated into English by accredited translators. Facilitators reviewed the translated transcripts for accuracy. When translations were unclear, facilitators revisited the recordings and made corrections directly to the transcript.

The study was approved by the European Food Information Council’s ethics committee (approval number: 2026/04/15/1, 15 April 2026).

### 2.3. Data Analysis

Data were analysed using thematic analysis in Atlas.ti Web (version 9.24.3) [30]. Two independent coders coded the English translations using an inductive (bottom-up) and deductive (top-down) approach. Codes were developed from the data and, initially, organised according to discussion topics and the resulting themes considered subsequently in relation to the adapted AAAQ framework. Coders met once every week during the coding process to discuss and resolve disagreements. Disagreements were resolved by explaining each coders reasoning and revisit the relevant data extracts to determine which interpretation is better supported. Once all transcripts had been coded and disagreements resolved, one coder (AP1, first author) identified themes and sub-themes, which were reviewed by the second coder (AP2, third author). A reviewer external to the coding team (BC) also reviewed the themes and subthemes, helping to ensure consistency and robust interpretation.

The agreed themes were organised into six domains. Adequacy was the overarching outcome domain capturing the extent to which participants obtained sufficient micronutrients, inferred from indicators such as reported consumption of micronutrient-rich and foods fortified with micronutrients, perceived dietary variety, reported use of food supplements, and perceived sun exposure (as a source of vitamin D). Perceived factors potentially affecting access to and consumption of micronutrient-rich foods were categorised using the adapted AAAQ framework.

Availability encompassed the presence and variety of micronutrient-rich foods in the local food environment and within food assistance programmes. Accessibility included aspects of physical (including access to food assistance, cooking facilities, social support networks, and sunlight), economic (e.g., affordability, price perceptions, financial constraints, welfare support, coping strategies), and temporal accessibility to micronutrient-rich foods. Accessibility to advice and information about micronutrients was also included in the Accessibility domain. Acceptability addressed personal and sociocultural factors that shaped willingness to consume micronutrient-rich foods and diets, including migration-related influences, mental health effects, and attitudes toward fortified foods and food supplements. Quality included perceptions about the quality and safety of micronutrient-rich foods and challenges related to food storage and preparation that were thought to compromise quality and micronutrient content. Finally, Knowledge and Perceptions about micronutrients were examined as a cross-cutting analytical domain, encompassing participants’ associations, knowledge, misconceptions, and beliefs about the roles and sources of micronutrients.

The frequency with which each code appeared in the analysis (i.e., number of quotations per code) was recorded, which allowed comparisons of themes between food assistance beneficiaries and refugees as well as between the two charitable organisations. An overview of the identified themes, subthemes, codes, and representative quotations is available in Supplementary File S1 in the Supplementary Materials.

## 3. Results

### 3.1. Participant Characteristics

A total of 68 people participated in the study: 26 in Trieste and 42 in Olsztyn. In Trieste, the two focus groups with food-assistance beneficiaries included eight and six participants, respectively, and the two focus groups with refugees included six participants each. In Olsztyn, the corresponding groups included ten and seven food-assistance beneficiaries and 13 and 12 refugees. Refugee participants in Olsztyn had mainly migrated from Ukraine and Belarus, whereas those in Trieste had migrated from countries in Europe, Asia and Africa.

The sample was predominantly female (81%), and participants ranged from 18 to 79 years of age. Just over half (51%) had completed upper-secondary education or below, while 46% had completed post-secondary or tertiary education; 3% preferred not to report their education. Reported employment circumstances included temporary unemployment (28%), manual or non-manual employment (25%), retirement (18%), inability to work, including for health reasons (12%), homemaking (9%), self-employment (7%) and study (6%) (participants could select more than one response). Most participants lived in urban areas (88%).

Annual household income was below €10,000 for 44% of participants, between €10,000 and €20,000 for 15%, and above €20,000 for 9%; 32% preferred not to report their income. Two-thirds (66%) reported that their household received neither social-security benefits nor disability income.

Compared with the sample in Trieste, the sample in Olsztyn comprised higher proportion of females, younger and middle-aged adults, highly educated participants, and lower proportion of older adults and those in the lowest income category.

Full participant characteristics by study site and for the overall sample are provided in Table S1 in the Supplementary Materials.

### 3.2. Adequacy

Participants talked spontaneously about their consumption of micronutrient-rich foods such as fruits, vegetables, meat, fish, dairy, legumes, nuts/seeds, and wholegrains during the discussions. Consumption of fruits and vegetables was mentioned frequently, while some participants in Poland, particularly food assistance beneficiaries, considered meat as essential or standard in the diet. A few participants reported avoiding or rarely consuming certain micronutrient-rich foods such as fruits, vegetables, pork, or dairy. Furthermore, very few participants, particularly refugees in Poland, intentionally consumed fortified foods, with most reporting that they did not consume them or were unsure whether they did. Participants also reported to what extent they incorporated variety in their diets. Narratives reflected variation, with some food assistance beneficiaries reporting having a varied diet, most participants describing their diets as insufficiently varied, and some reporting efforts to modify their diets by, for example, reducing meat intake or consuming more vegetables or legumes. Regarding sun exposure, most participants reported at least 10 min per day, while many reported having vitamin D deficiency. Deficiencies in iron, magnesium, potassium, selenium, calcium, zinc, and B-vitamins were also reported by participants, based on their recollection of clinical test results or consultations with doctors. Participants frequently reported using food supplements in response to deficiencies or bodily symptoms (e.g., fatigue, cramps) or used them preventively, for example, to compensate for an inadequate diet, recover micronutrients they believed were lost during cooking, maintain general health, or meet micronutrient needs during pregnancy. Many participants did not report specific reasons for using food supplements; others mentioned not using food supplements.

### 3.3. Availability

Participants expressed mixed views on the availability of micronutrient-rich foods in their environments. Changing weather patterns, seasonality, environmental pollution, and food system disruptions (e.g., due to war) were discussed by participants as factors perceived to limit the availability of micronutrient-rich foods, particularly fruits, vegetables, and fish, in broader food environments. In Italy, food assistance beneficiaries viewed increased reliance on imports as both compensating for perceived climate-related shortages and undermining local production. As one participant explained, “That is, we discard our products because they are poorly paid and obviously the farmers lose us as an income source and therefore sometimes they do not even take them to the market because they want to pay the [inaudible], while they import all the stuff from abroad with all the pesticides and garbage that are grown” (P20, food assistance beneficiary, Italy). When it comes to local retail environments, many described high availability of micronutrient-rich foods, citing well-stocked stores providing diversity, foods from around the world, and organic products. Others, mainly participants in Poland, reported limited or unstable availability, including fluctuating supermarket stock levels during the day, unpredictable availability in open markets, regional differences in the availability of certain foods like fish, and lack of food provision or limited meal options in workplaces, which limited opportunities to obtain and consume micronutrient-rich foods. Mixed views were also expressed by refugees in Poland regarding the availability of micronutrient-rich foods compared with their home country and the availability of food aid when they arrived in Poland. Some considered the support they received adequate, noting that meals were diverse and often included a good range of micronutrient-rich foods, while others received insufficient food quantities to cover their needs. Views also diverged regarding the availability of fruits and vegetables in food aid.

Availability was also discussed in the context of food assistance. Food assistance beneficiaries in Trieste emphasised the limited availability of micronutrient-rich foods in the social supermarket, particularly fruits, vegetables, and meat. Some reported that availability in rural social supermarkets was better than in urban social supermarkets, which they attributed to procurement agreements with local suppliers. Food assistance beneficiaries in Olsztyn, in contrast, reported high availability of micronutrient-rich products in the food bank, including canned legumes, meat, fish, and fruit. Quantity and variety perceptions also varied. Some considered quantities insufficient to meet household needs or found the variety insufficient to support a balanced diet, while others were satisfied with these aspects. Participants in Poland additionally noted that availability fluctuated with donation patterns, at times resulting in excess of some products.

Overall, participants’ accounts suggest that the availability of micronutrient-rich foods is context-dependent and uneven across food environments, geographical locations, and sources of food provision. Participants believed availability is shaped by broader environmental and food system factors, as well as by local procurement and donation practices, contributing to variation in the quantity and variety of micronutrient-rich foods available to different groups.

### 3.4. Accessibility

#### 3.4.1. Physical Accessibility

Participants described mixed experiences regarding the physical accessibility of micronutrient-rich foods. Some stated that it was easy to find such foods as stores were conveniently located in their area and online shopping was also available for those who lack time for store visits. However, others reported difficulties, in both urban and rural areas, especially when it came to micronutrient-rich food that was perceived to be more affordable, of better quality, or natural/less processed. As one participant explained, “It’s difficult. For me, coming from the countryside, is very difficult, because things are done differently in the countryside, food isn’t natural, let’s say, it’s more processed, especially meat, even with vegetables. It is difficult to find 100% natural and healthy stuff” (P01, food assistance beneficiary, Italy). Barriers included long distances to affordable or good quality stores, lack of transportation, cost of commuting on public transport, and logistical challenges such as carrying groceries on foot or weather-related constraints. Restricted accessibility to micronutrient-rich food in workplaces was also noted, policies restricting employees from bringing home-prepared food to work, limited availability of stores nearby, lack of (or insufficient) equipment to store and reheat food, or stressful work environments that make it hard to leave the premises for lunch. Work environments that promoted access to micronutrient-rich food were reported infrequently.

Refugees (mainly in Poland) further discussed how migration affected their accessibility to micronutrient-rich foods, with some reporting similar or improved accessibility in the host country (e.g., regarding dairy and international foods). Others reported lower accessibility either due to absence of social support networks (e.g., food sharing among relatives) or loss of food production opportunities (e.g., home or allotment gardens). As one participant noted, “Well, let me say that we had ours there, and here only from the store. And it really makes a big difference” (P59, refugee, Poland). Refugees and food-assistance beneficiaries in Italy described social isolation within urban communities as a barrier to accessing micronutrient-rich food. Furthermore, refugees in Poland living in temporary accommodation (hostels) encountered challenges with restricted access to cooking facilities/equipment, including scheduled access to shared kitchens and insufficient space to cook, which reduced their ability to prepare nutrient-dense meals. Likewise, refugees in Italy who were living on the premises of charitable organisations described having to adjust their eating habits to comply with accommodation policies that prohibited bringing in or preparing their own food. As one participant noted, “The will is very much there, but I can’t do anything, because it can’t be done. We all eat what they give us to eat” (P23, refugee, Italy). Participants living independently reported greater autonomy over food choices but also described reduced food affordability because of rental costs.

Problems with accessibility of food assistance/aid services were also discussed. Food assistance beneficiaries in Trieste reported that visit quotas limited their access to the social supermarket. Alongside the limited availability of micronutrient-rich foods in the social supermarket, visit quotas constituted a substantial barrier to accessing these foods through food assistance in Trieste. In contrast, a participant in Poland mentioned additional community initiatives including community fridges, which provided access to food alongside the food bank in Olsztyn. Experiences diverged also with respect to food aid, with some refugees reporting access to food aid from charitable organisations and churches that facilitated consumption of micronutrient-rich foods, while others reported experiencing delays or received no food aid at all, sometimes due to not actively seeking help.

Finally, participants discussed factors influencing their access to sunlight in relation to vitamin D synthesis. Sedentary lifestyles, time constraints such as working during daylight hours, limited sunlight associated with latitude or frequent bad weather, mentioned particularly in Olsztyn in northern Poland, and heavy clothing were believed to limit sun exposure. Some participants reported low sun tolerance or expressed health concerns related to sun exposure (e.g., ageing skin, skin cancer, dizziness, vision problems, interactions with medication). These concerns made some participants reluctant to seek intentional or prolonged sun exposure as a means of increasing vitamin D synthesis. Beliefs that vitamin D primarily comes from food rather than being synthesised in the skin following sunlight exposure were also expressed. Yet, participants also identified factors that increased sun exposure, such as enjoying sunny weather, walking as a means of transport, outdoor occupations, and outdoor leisure activities including spending time outside with children or pets.

#### 3.4.2. Economic Accessibility

Participants widely reported economic hardships and highlighted trade-offs between food and other essential expenses, such as rent, bills, medical costs associated with accidents or disabilities, or costs for supporting dependent family members. One refugee in Italy perceived foreign nationals as having less financial stability than permanent residents. Participants described unemployment and dependence on limited pensions or social welfare as exacerbating financial strain. Some described limited access to social welfare, even in circumstances they considered indicative of clear need, such as single parenthood. Likewise, refugees in Poland mentioned restricted access to financial aid. Among those who were receiving some kind of financial support, some felt the support was insufficient, explaining that payments were too low to cover basic needs or remained unchanged despite inflation. Some participants expressed a sense of injustice toward the welfare system, feeling that eligibility criteria were sometimes unfair or too strict. Food assistance beneficiaries in Trieste further described experiences they regarded as institutional neglect, including limited communication from authorities, insufficient engagement from social workers, and feeling that their nutritional concerns were being dismissed. Stigma also surfaced, with some food assistance beneficiaries in Trieste expressing discomfort about accepting social assistance or asking their children for financial help. In contrast, participants in Poland mainly conveyed appreciation for the financial support they received, noting that it helped them purchase micronutrient-rich foods.

Given widespread financial hardships, most participants reported limited affordability of micronutrient-rich foods, highlighting high prices for fish/seafood, fruits, vegetables, meat, and other protein sources such as legumes, nuts/seeds, and dairy products. Participants perceived inflation as particularly affecting people reliant on smaller or rural stores, which they considered more expensive than supermarkets. Participants also associated higher prices with reduced availability, for example, because of seasonal, climate-related, or pollution-related shortages. For some participants, high prices meant that they rarely purchased certain micronutrient-rich foods such as cheese, fish, or certain fruits (e.g., kiwi). Additionally, budget constraints often limited dietary variety, with participants prioritising cheaper, less nutrient-dense options over a more diverse intake of micronutrient-rich foods. As one participant noted, “Sometimes it’s also a financial problem. Because to spend less perhaps you buy all the things that cost less but are worse for you. So you don’t go and buy meat, fish… All things that cost more. So it all has a knock-on effect, unfortunately, right?” (P04, food assistance beneficiary, Italy). Yet, a few felt that micronutrient-rich foods, such as fruits, vegetables, eggs, or fatty fish, were affordable, indicating variation in affordability perceptions among participants. Refugees in Poland also had mixed opinions about the affordability of micronutrient-rich foods compared to their home country, with some perceiving similar or greater affordability in the host country, noting cheaper fish, vegetables, and generally improved finances, while others reported reduced affordability due to new financial obligations (paying for rent), lack of space to grow food, or specific items, such as beef, being more expensive.

To cope with limited budgets, participants reported using a wide range of financial, shopping, food preparation, and dietary coping strategies. Financial strategies included cutting unnecessary purchases, prioritising essential expenses, setting daily food budgets, or seeking financial help from neighbours, relatives, or charitable organisations. When it comes to shopping, many engaged in discount-hunting, using apps (e.g., to purchase food close to the expiry date at a discounted price), store hopping, or shopping at discount supermarkets. Others focused on purchasing the cheapest options through buying in-season produce, buying by weight, and travelling or even crossing borders to find more affordable stores. Budget-conscious shopping practices, such as using shopping lists, reducing store visits, avoiding shopping on an empty stomach, or planning purchases according to available budget, were common. Participants reported that meal planning helped reduce unnecessary spending and was convenient when preparing multiple meal variations to satisfy diverse preferences in the household, thereby also facilitating acceptability. Furthermore, some individuals made trade-offs between quality and price, either buying smaller quantities of foods they perceived as higher quality, opting for budget-friendly products they perceived as lower quality, or prioritising key products based on nutritional value (e.g., grains, protein sources). Other strategies included shopping on credit, obtaining food from food assistance, or prioritising needs of certain family members when shopping, especially children or elderly people. Beyond conventional shopping, some participants also mentioned growing their food (e.g., in gardens, allotment spaces, or balconies) or foraging/hunting. However, some noted that these practices can involve risks, and require specific knowledge. Participants also maximised value through food preparation strategies. Batch cooking or buying in bulk and freezing or canning helped, according to participants, extend the lifespan of perishable foods and save money, although some noted that canning required time and individual effort. Participants also described being resourceful in the kitchen, such as investing time in cooking, using available ingredients creatively, repurposing canned foods obtained from food assistance, making homemade versions of typically store-bought foods, and following zero-waste principles to minimise waste and maximise value. Finally, dietary coping strategies were also reported, mostly by refugees. These included substituting legumes, vegetables or eggs when meat or fish were too costly; self-restriction, such as skipping meals or reducing portion sizes; seeking satiety through eating healthy food, which participants thought kept them full for longer; drinking water; or reducing variety by eating the same meals over consecutive days. During times of scarcity, some family members, particularly children and elderly people, were given priority in food allocation.

#### 3.4.3. Temporal Accessibility

While cooking was thought to facilitate the consumption of micronutrient-rich foods in a more affordable way and promote dietary variety, time constraints were identified by some as a barrier to cooking. However, a few participants challenged the assumption that preparing less healthy food was necessarily faster, indicating that time was not universally perceived as a barrier to preparing micronutrient-rich food. Furthermore, participants sometimes struggled to fit grocery shopping into demanding work schedules or found it hard to invest the extra time needed to visit stores they perceived as higher quality, which were often farther away, thereby resorting to more convenient stores they perceived as lower quality. Working participants in Poland felt that the window for shopping was restricted for people who work, noting that by the time they reached shops after work hours, the availability of fresh or high-quality products was noticeably lower. These time constraints were perceived as a barrier to regularly purchasing micronutrient-rich foods, particularly fresh items. Furthermore, participants reported that time pressures at work such as short or infrequent breaks, strict schedules, limited opportunities to eat before shifts, or insufficient time to prepare food at home, meant that participants often skipped meals or relied on quick, convenient options rather than nutrient-rich meals. Finally, some refugees reported that changes in their work situation after arriving in the host country, including a transition from not needing to work previously, limited the time available to obtain and consume micronutrient-rich food.

#### 3.4.4. Information Accessibility

Participants received advice about micronutrients from health professionals (i.e., mainly doctors but also dietitians, pharmacists, etc.), media sources (e.g., news outlets, ads, tv, internet), interpersonal networks (e.g., friends, colleagues, word of mouth), or artificial intelligence tools, although some did not seek advice at all. Some participants reported limited access to professional guidance. For example, food assistance beneficiaries in Trieste faced difficulties scheduling in-person consultations with doctors specifically about micronutrient issues and reported receiving food supplement prescriptions remotely without further guidance or receiving advice only after deficiencies had been identified. In turn, refugees in Poland expressed uncertainty about where to get tested for micronutrient deficiencies or how much testing would cost. Advice participants had received was primarily concerned with food sources to tackle micronutrient deficiencies (mainly in Poland) or with food supplement use. Some participants reported high adherence to advice from doctors, while others encountered difficulties following dietary recommendations or trusting advice from artificial intelligence or word of mouth.

Participants also cited diverse channels they might use to obtain information about micronutrients, including formal sources (e.g., government websites, books), interpersonal channels (friends), social media, traditional media, and self-directed digital channels (e.g., internet, artificial intelligence). They discussed barriers to navigating the information landscape, mainly in Poland, specifically information overload, conflicting messages, sources they considered unreliable, poorly communicated or fragmented information, as well as limited time and motivation to stay informed. Limitations of professional guidance were also discussed, including perceptions that dietitians provided overly generic information or lacked practical knowledge about food technology or cooking, and that dietary recommendations changed over time, which some participants said reduced their trust in the guidance.

While some were not interested in receiving more information about micronutrients, many would welcome clear, personalised, and actionable information, particularly on food sources of micronutrients, including budget-friendly food alternatives or guidance on how to maximise micronutrient delivery with food combinations or cooking techniques that preserve nutrients, food supplements (e.g., dosage, length of use, interactions, effectiveness), testing for micronutrient status (mainly refugees in Poland), or deficiency symptoms. Others would be interested in general dietary recommendations. Finally, refugees in Poland additionally suggested systemic solutions beyond receiving information, such as easier access to testing for deficiencies (e.g., free annual or biannual deficiency tests), meal provision services (e.g., nutrient-rich and tasty meals designed by dietitians and culinary professionals), or technological solutions that could assist health professionals in providing advice about micronutrient deficiencies including what one participant described as “deficiency-detecting chips”.

Overall, participants’ accounts indicate that accessibility to micronutrient-rich foods is multidimensional, with physical, economic, temporal, and informational barriers often interacting and compounding one another. Physical and financial constraints could limit access to food outlets that were perceived to be of better quality of more affordable, while limited time and restrictive work or living arrangements further constrained opportunities to shop for, prepare, and consume micronutrient-rich foods. Challenges with accessing professional advice and navigating the complex food information environment further undermined participants’ ability to make informed decisions about meeting their micronutrient needs. At the same time, participants described a range of adaptive strategies to overcome accessibility barriers, although these strategies often required additional time, resources, knowledge, or social connections.

### 3.5. Acceptability

Some participants reported liking micronutrient-rich foods such as fruits, vegetables, meat, dairy, and fish, while others described clear dislikes that limited intake of some food groups. In most cases, dietary restrictions due to allergies, intolerances, or health conditions, limited the range of micronutrient-rich foods participants could consume, although one participant mentioned trying to cope with a health condition (psoriasis) by replacing junk food with fruits and vegetables. Dietary restrictions, food preferences (e.g., stronger preference for foods perceived as less nutritious), and regarding eating as a chore or hassle also limited dietary variety. Participants also discussed the role of habit, reporting that micronutrient-rich foods became more acceptable as they became more familiar and highlighting the importance of being exposed to new flavours, especially among children.

Culinary practices influenced the acceptability of micronutrient-rich foods. Participants in Poland noted that adding flavourings, combining foods to improve taste, or using preservation methods can make otherwise bland foods (e.g., vegetables) more appealing. Cooking was also discussed in the context of dietary variety. Some viewed cooking as a barrier to eating a varied diet (e.g., due to lack of energy, motivation, or knowledge to cook), while others discussed how cooking facilitates variety (e.g., cooking healthy versions of fast foods at home). Culinary practices that were thought to promote the consumption of micronutrient-rich foods and diversify the diet included cooking from scratch, incorporating vegetables, fruits, seeds, or legumes into meals covertly, or making micronutrient-rich foods visually appealing to encourage children’s acceptance.

Social environments also shaped acceptability and consumption of micronutrient-rich foods. Supportive environments were characterised by spouses, children, and extended family members who encouraged healthier eating, shared cooking responsibilities, or welcomed nutritious meals. Food assistance beneficiaries in Trieste discussed how the wider community, including neighbours and friends, facilitated the consumption of micronutrient-rich foods by sharing home-cooked meals and providing support in times of scarcity. In contrast, non-supportive environments were characterised by conflicting food preferences within the household (e.g., partners favouring unhealthy foods, multi-generational households with divergent habits) and dietary restrictions or resistance to new flavours among family members, especially children, which often restricted the inclusion of micronutrient-rich foods in family meals. Furthermore, some participants encountered social pressure from relatives or friends to eat unhealthy foods or described broader social norms and media environments that they felt undermined healthy eating, which posed challenges to selecting nutrient-rich options. Thus, overall, participants described both supportive and constraining influences from their social networks, while some perceived no social influence on their diets.

Refugees described varied effects of migration on their eating habits. For some, migration brought little or no change, sometimes due to similarities in food traditions and environments between home and host country. Others reported a positive fusion of host- and home-country culinary practices or mentioned improvements in their diets such as eating a more plant-forward and less meat-heavy diet, which could enhance the breadth of micronutrients obtained from food. Yet, several participants experienced challenges with the cultural compatibility of food in the host country, noting differences in commonly consumed meats (e.g., pork eaten in the host country but not the home country), lack of Halal options, or culturally incompatible food being offered in food aid or workplaces (mainly in Poland), leading to avoidance of meals in those settings.

Cultural and religious food traditions also influenced the consumption of micronutrient-rich foods for both refugees and non-refugees, though their influence varied. Food assistance beneficiaries in Olsztyn discussed how national food traditions and historical experiences affected how they navigated their diets. For example, some described prioritising having enough food as a legacy of post-war Polish food culture, but it was unclear whether their accounts focused primarily on having sufficient food or on its nutritional quality. Religious food traditions (mainly related to Catholicism) usually involved abstaining from specific micronutrient-rich foods (mainly meat or dairy) on certain days/holidays. In some cases, however, food traditions facilitated the consumption of micronutrient-rich foods (e.g., eating fish on Ash Wednesday). Participants in Poland also mentioned intergenerational food traditions, with recipes, cooking skills, and food attitudes passed down within families, sometimes supporting healthier choices and sometimes constraining them. Some participants, however, reported no cultural or religious influence because food traditions were hard to follow with limited budgets, were perceived as irrelevant, or were gradually fading.

Furthermore, although some participants reported no influence of mental health on their dietary choices, most associated poor mental health with reduced consumption of micronutrient-rich foods. Participants reported that stress, anxiety, and depression were associated with food choices they considered less healthy and overeating (e.g., increased consumption of sweets, reliance on junk food over home-cooked meals), reduced dietary variety, or alcohol consumption. Participants in Poland, however, also reported experiencing loss of appetite, sometimes to the extent of avoiding food altogether. Refugees linked mental health difficulties to their experiences of war and displacement but reported that preparing and consuming nutritious food improved their mental well-being.

Regarding fortified foods, most participants held negative attitudes, driven by disinterest, scepticism, or a stronger preference for non-fortified foods. Many viewed fortified foods as unnecessary, thinking that normal foods already provide the necessary micronutrients. Distrust was common, with some perceiving fortification as a trend or a marketing tactic that increased the cost of foods. Others expressed scepticism about processing of fortified foods, including beliefs that they contain harmful additives or artificial vitamins, or that fortification spoils the food. As one participant explained, “The most important vitamins are the living ones. Just like in vegetables or fruit, and not some artificial products, because it’s chemistry” (P36, food assistance beneficiary, Poland). Many preferred to cover micronutrient needs through non-fortified foods, particularly fruits and vegetables, which were viewed as more reliable sources of micronutrients than fortified products. These negative beliefs were reported alongside low intentional consumption of fortified products.

Finally, attitudes toward food supplements ranged from rejection or strong scepticism to endorsement. Many described food supplements as expensive, artificial, unnecessary, ineffective, or viewed them as a marketing trick or a passing trend, and preferred meeting micronutrient needs through food. Some expressed scepticism toward marketing of food supplements by both social media users and doctors. Participants in Poland expressed concerns about insufficient clinical testing, limited quality control, and side effects of food supplements or reported low tolerance (e.g., due to difficulties swallowing pills). Some viewed food supplements as necessary, as they believed food alone cannot fully cover micronutrient needs or even treated them as a replacement for food. One participant mentioned that food supplements contain fewer chemicals than food preservatives. Others considered food supplements helpful or convenient, or emphasised that they should be used only when advised by a doctor, not in excess, or in a personalised way (e.g., depending on lifestyle and specific physical needs). Furthermore, participants recognised differences between food supplements. Food assistance beneficiaries in Olsztyn distinguished food supplements prescribed by doctors from commercially available food supplements, perceiving prescribed food supplements as more effective, more consistent in dosage, and safer. As one participant explained, “This is not supplementation. Because they are not supplements, they are prescribed drugs. This is not a supplement. I supplemented with vitamin D3 for 10 years and it rose by 2, 3 units only. How many of these pills can we eat? How do we damage the liver? From 25 it rose to 28, and here one dose in a year of the drug, which cost 60 zlotys. It is much cheaper, only the doctor has to prescribe it. In my case it rose from 25 to 50 and it stays on that level for six months” (P37, food assistance beneficiary, Poland). They also described injections as preferable to tablets because they believed injections offered better absorption or longer-lasting effects. Participants in Italy, by contrast, reported that they believed food supplement quality depended on the brand, although some believed that active ingredients were similar across brands.

Taken together, the findings indicate that the acceptability of micronutrient-rich foods was shaped by the interplay of individual preferences and restrictions, culinary practices, family dynamics, social and cultural environments, and mental well-being. Participants’ accounts also highlighted a strong preference for obtaining micronutrients from conventional foods, alongside considerable skepticism towards fortified foods and divergent attitudes towards food supplements.

### 3.6. Quality

Participants were asked about the quality of micronutrient-rich foods in broad terms, rather than specifically in relation to nutrient density. As such, they perceived quality in diverse ways including naturalness, tastiness, freshness, safety, credence labels, and micronutrient content. Participants considered food quality important but perceived a decline in quality and naturalness over the years, noting, for example, that fresh produce lacked taste due to early harvesting or that food had become more and more artificial and less nutritious. Food safety concerns related to increased pesticide use and pollution were also voiced among participants in Italy. As one participant noted, “It isn’t natural. Now. There are so many pesticides. I don’t think there are as many vitamins in food as there used to be” (P14, refugee, Italy). Refugees described contrasting experiences with food quality when comparing their home and host countries, with some perceiving higher quality in the host country and others criticising overly processed foods, tasteless fruits or meat, or misleading credence labels (e.g., ‘natural’, ‘local’, ‘homemade’) in the host country. Perceived quality further depended on the food provisioning environment. Home-grown and local products were perceived as more genuine, lower in pesticides, and of superior quality, and open markets were often considered as offering higher quality than supermarkets or discount stores, though some raised food safety concerns due to inappropriate storage practices in open markets. Although participants perceived food quality through multiple dimensions that do not necessarily reflect micronutrient content, some indicated that negative perceptions of the quality of micronutrient-rich foods reduced their willingness to obtain or purchase them, even when they were accessible.

Food quality in organisational settings (e.g., food assistance, workplaces) was also discussed. Participants who received food at work generally described its quality negatively. Likewise, most food assistance beneficiaries in Trieste perceived the quality of food provided through food assistance as low, noting that products were often imperfect, near expiry, or characterised them as ‘second-rate’. While these perceptions reduced willingness to purchase certain micronutrient-rich foods among some beneficiaries, others said that they were not affected because they were accustomed to eating such food. In contrast, most food assistance beneficiaries in Olsztyn were satisfied with the quality of products offered by the food bank, reporting that donations sometimes matched or even exceeded supermarket standards.

Participants also identified links between quality, availability, and affordability of micronutrient-rich food. Some participants felt that off-season produce is artificial, tasteless, and lower in vitamins (content), and perceived year-round availability as coming at the expense of quality. As one participant noted, “I’ve noticed that now you can find everything, all year round. Then what you get is low-medium quality. I don’t know how good the nutrients can be” (P06, food assistance beneficiary, Italy). In addition, some participants highlighted a trade-off between food quality and affordability, noting that foods they perceived as high quality were more expensive or, conversely, discount products were perceived to have lower vitamin content. Some participants described this perceived trade-off as influencing the foods they selected within their available budgets. However, others were sceptical of the price-quality link, noting that consumers have no guarantee that more costly items are of better quality.

Furthermore, participants discussed aspects of food storage and preparation (e.g., improper storage temperatures, storage length, damaged or inappropriate packaging) as perceived determinants of food quality and nutrient retention, alongside challenges they faced in managing food under their living conditions. Food preparation, processing, and cooking (e.g., cutting or handling foods improperly, high-temperature cooking, frying, overcooking) were believed to reduce micronutrient content and affect tolerance or absorption in the body, whereas freezing was perceived ambivalently: some believed it preserved freshness without reducing nutrients, while others thought that freezing, especially for long periods, altered food quality or even destroyed vitamins. These accounts reflect participants’ beliefs that attributes such as freshness and minimal processing are directly linked to higher micronutrient content.

While some participants did not encounter any challenges with food storage and preparation, others described barriers that affected how they managed micronutrient-rich foods. For example, limited storage capacity (e.g., having no refrigerator or only a small one) was perceived as restricting the ability to store micronutrient-rich foods, leading participants to adopt adaptive strategies to maintain freshness such as storing items outdoors during winter, keeping food in cold water, or consuming everything quickly. Other strategies to maintain freshness included cooking daily, avoiding stocking up food, managing storage to facilitate first-in-first-out use of perishables, using coolers to transport food after shopping, and freezing. However, participants from larger households reported such practices were sometimes challenging because of difficulties with purchasing fresh food daily or keeping track of stored food.

These findings indicate that participants understood quality of micronutrient rich foods as a multidimensional concept, encompassing freshness, taste, naturalness, safety, credence attributes, seasonality, and perceived micronutrient content. These dimensions were closely interconnected with availability and affordability perceptions and influenced purchase decisions. Finally, storage, preparation, and cooking practices were viewed as important for maintaining food quality and micronutrient content. Overall, the findings suggest that the quality dimension extends beyond the nutritional composition of foods to encompass how foods are perceived, handled, stored, and prepared within different food environments.

### 3.7. Knowledge and Perceptions

When asked about the first thing that comes to mind when thinking about vitamins and minerals, most participants mentioned food sources of micronutrients (e.g., fruits, vegetables, meat, fish), although other associations were also evident such as with specific vitamins, health benefits (e.g., strength, healthy sleep), broader notions of healthy lifestyle (e.g., healthy eating, diet), food supplements, deficiencies, or medical settings (e.g., hospital, pharmacy). Familiarity with the term ‘micronutrients’ varied widely. Many were unfamiliar with the term, and some refugees encountered linguistic difficulties with understanding or explaining the term. Misconceptions were also common, including incorrect beliefs about what micronutrients are (e.g., mixing up micronutrients with microbes or perceiving micronutrients as elements with minimal nutritional value), which nutrients count as micronutrients (e.g., believing that omega fatty acids are micronutrients), and how deficiencies or toxicity develop. Examples included beliefs that micronutrient deficiencies inevitably develop at some point in life or always produce recognisable bodily symptoms, that eating too many carrots causes vitamin A toxicity, and that excessive intakes of magnesium or vitamin D cannot readily cause harm. Some expressed partial or simplified understanding, stating that, for instance, micronutrients are food supplements, are found in fruits and vegetables, or are small components of food, without further elaboration. Others demonstrated a more accurate understanding, recognising that micronutrients include vitamins and minerals, are required in small amounts in the body, are found in ordinary food, or that everyone can be vulnerable to micronutrient deficiencies.

Despite these varied levels of familiarity with the term, participants widely recognised the essential role of vitamins and minerals for the body and linked them to the proper functioning of various body systems (e.g., immune, skeletal, nervous, digestive, cardiovascular, endocrine, muscular, integumentary), physiological processes (e.g., body restoration and strength, vision, brain and cognitive function), and mental health (e.g., depression), or discussed their importance for development and general well-being. Participants frequently linked specific vitamins or minerals to functions (e.g., calcium for the bones, vitamin C for the immune system), dysfunctions (e.g., vitamin A deficiency causing poor eyesight), or nutritional deficiency diseases (e.g., scurvy caused by vitamin C deficiency) and also mentioned toxic effects (e.g., of fat-soluble vitamins).

Finally, participants identified a wide range of food sources of micronutrients, most commonly fruits and vegetables, followed by meat, but also other food groups such as fish, dairy, nuts/seeds, legumes, plant-based fats, and mineral water. Some referenced fermented vegetables, fortified foods, farm-grown meat, naturally grown vegetables, or raw vegetables, indicating that they associated micronutrient content with food-processing or production methods. Others mentioned non-food sources, including sunlight exposure in relation to vitamin D synthesis and the use of food supplements. Dietary diversity was also acknowledged as an important factor for obtaining micronutrients. While discussing micronutrient sources, some participants in Poland highlighted uncertainties around micronutrient content and bioavailability of food (e.g., what quantities we get from the amounts of food we typically consume), which they felt limited their ability to make informed decisions about including micronutrient-rich foods in the diet. Participants also discussed antagonistic/facilitating effects between foods and nutrients, including beliefs that legumes reduce micronutrient absorption, coffee depletes micronutrients, and dietary fat is required for certain vitamins to be absorbed.

Overall, the findings highlight a distinction between awareness of the importance of micronutrients and the more specific, actionable knowledge required to translate this awareness into informed dietary practices that support micronutrient adequacy. An overview of the main barriers to micronutrient adequacy reported in this study can be found in Figure 1.

## 4. Discussion

This pilot study examined the diverse and interacting factors perceived to compromise access to and consumption of micronutrient-rich foods and potentially increase vulnerability to micronutrient deficiencies among food assistance beneficiaries and refugees in Italy and Poland. Participants’ accounts of constrained consumption of micronutrient-rich foods, limited dietary variety, rare deliberate use of fortified foods, and food supplement use in response to diagnosed or suspected deficiencies indicate difficulties in accessing and consuming varied, micronutrient-rich diets (i.e., dietary patterns that provide adequate intake of micronutrients and reduce the risk of micronutrient deficiencies). However, micronutrient adequacy was not directly assessed. Across the domains of the AAAQ framework, personal, interpersonal, contextual, and structural factors interacted to shape participants’ access to and consumption of micronutrient-rich foods. The identified barriers align closely with those reported in prior research for groups with socioeconomic disadvantage. For example, budgetary constraints represent a central barrier to accessing nutritious foods among food assistance beneficiaries [10,11] and refugees [31] in previous studies. Low availability of nutrient-dense food in socially deprived areas [32] and within charitable food services [11,33], time constraints [10,11,34], transportation difficulties [9,34], and challenges with family food dynamics [35,36] have also been previously reported. Consistent with the present findings, previous studies have also identified knowledge gaps related to micronutrients, including limited awareness about recommended intakes of vitamin D and calcium, their interactions with other nutrients, and risk factors or consequences of deficiencies [24], particularly among individuals with lower educational attainment and income [37]. Finally, consistent with emerging evidence [38], participants in this study associated mental health challenges, including anxiety and depression, with reduced consumption of micronutrient-rich foods. These accounts might be consistent with proposed biobehavioural and psychological responses to scarcity, including stress and reduced cognitive capacity, that can affect dietary decision-making [39].

While the barriers identified in this study could individually affect access to and consumption of micronutrient-rich foods, participants’ accounts suggest that overlapping barriers might have greater consequences. Although most participants expressed a preference for meeting their micronutrient needs through food rather than food supplements, not all food sources of micronutrients were perceived equally. Participants expressed more positive attitudes towards natural, minimally processed food sources, as opposed to, for example, fortified foods, which were viewed as more artificial, costly, and less reliable for micronutrient delivery. Moreover, a preference for fresh, home grown, and local foods was evident, as these attributes were perceived as indicators of better quality. These findings are supported by prior evidence indicating a preference for natural, unprocessed, fresh products among refugees in European countries [31,38]. These preferences could, nevertheless, constrain participants’ choices, since foods perceived as high quality were also regarded as expensive and hard to access. Combined with acceptability barriers within participants’ environments (e.g., rejection of certain food groups by family members), these tensions could weaken both their motivation and capacity to select and consume micronutrient-rich foods when resources were limited.

Furthermore, these dynamics might more severely undermine the consumption of specific types of micronutrient-rich foods than others. For example, participants frequently discussed barriers to accessing and consuming fruits, vegetables, fish, and meat, such as high cost of these foods [40], limited acceptability due to personal/family dislikes or variability in palatability (e.g., linked to seasonal variations for fresh produce or quality variations for fish/meat), less stable availability (e.g., susceptibility of fresh produce to weather challenges, regional differences in fish availability), or difficulties accessing higher quality stores offering these products. These findings align with previous research showing that refugees who experience food insecurity have a lower intake of fruits, vegetables, and fish than food secure refugees [14], while findings for meat are less consistent [41,42]. Barriers affecting these food groups can reduce access to important dietary sources of nutrients such as vitamin C, folate, and iron. However, the present study did not assess nutrient intake or micronutrient status and therefore cannot determine whether participants had inadequate intakes or were at increased risk of deficiencies. Previous studies have nevertheless reported a high prevalence of low vitamin C, folate, and iron intakes or status among socioeconomically disadvantaged groups [6,15].

The study also observed divergent attitudes toward food supplements. Previous research has found that lower income and education groups are less likely to use food supplements [43,44], a difference that has been attributed to knowledge, affordability, accessibility, health concerns, and personal preferences [45]. The present findings do not allow food supplement use to be compared across income groups, but they identify similar barriers and suggest that distrust concerning effectiveness, marketing, clinical testing, and quality control might also influence participants’ attitudes towards food supplements. Nevertheless, some participants viewed food supplements as necessary or substitutes for food. These perceptions might partly reflect reported limited access to professional guidance on micronutrients and emphasis on food supplements in some of the advice they had received. Taken together, these results underscore the need for improved, evidence-based communication about appropriate food supplement use, including the circumstances in which micronutrient needs should primarily be met through food and those in which professionally advised supplementation might be appropriate.

Furthermore, the study identified differences between the two study settings, which might reflect differences in local food environments, welfare arrangements, information environments, and organisational characteristics (i.e., service provision by Caritas Trieste versus Food Bank in Olsztyn). In Trieste, participants raised concerns about broader food system dynamics and food safety, which they believed undermined availability, affordability, and quality of micronutrient-rich foods. They also discussed about institutional neglect, stigma, and social isolation, which were thought to constrain access to nutrient-dense diets. These challenges were compounded by the limited availability, accessibility, and perceived low quality of charitable food assistance, as well as the restricted access to professional advice on micronutrient intake. Food-assistance beneficiaries in Olsztyn generally described the food bank as facilitating access to micronutrient-rich foods. However, participants in Olsztyn also reported limited availability and accessibility of foods that were affordable or perceived as high quality in retail settings, which they associated with demanding work schedules and time constraints. Other challenges discussed particularly in the Olsztyn groups included difficulties navigating information about micronutrients and perceived limitations on sun exposure associated with geographical location and weather. These findings underscore the need for tailored approaches to tackle challenges to micronutrient adequacy identified in each setting. Yet, it is important to note that sociodemographic differences were also observed between the Trieste and Olsztyn samples, with the latter comprising more females and younger, more highly educated participants, and fewer participants in the lowest income category. These differences may have contributed to variation in the experiences and perceptions reported across settings and may partly explain the observed differences alongside contextual factors. This research also advances understanding of the challenges encountered by refugees in accessing and consuming micronutrient-rich foods, a largely understudied area. Previous research has found that financial constraints, language barriers, unfamiliarity with host-country food systems, lack of cooking skills and food storage/preparation infrastructures, limited access to culturally appropriate foods, and social isolation are key barriers to healthy eating for refugees in Switzerland [31]. Furthermore, challenges with insufficient and culturally incompatible food aid along with inadequate cooking facilities have been reported for refugee shelters in the European context [13]. Our results are consistent with these findings, as participants described restricted access to financial aid, insufficient food aid, restricted access to food or cooking facilities in temporary shelters, provision of culturally incompatible food in organisational settings, poor mental health linked to displacement and war experiences, and unfamiliarity with local healthcare systems as barriers to accessing and consuming micronutrient-rich foods. In addition, we found that several refugees perceived the host country as offering lower availability, accessibility, affordability, and acceptability (taste) of micronutrient-rich foods compared with their country of origin, particularly when they had previously relied on home food production. In line with these findings, a study with refugees in the Netherlands showed that refugees perceived certain food groups (i.e., fruits, vegetables, meat, fish) available in the host country to be less tasty than corresponding foods from their countries of origin [36]. Yet, that study also found that traditional, culturally appropriate, and host-country foods were perceived as readily available in the host country, illustrating that experiences and perceptions of food availability might vary across populations and settings. Given these constraints, refugees in our study reported limited dietary variety and the use of dietary coping strategies during periods of scarcity, such as food restriction or intra-household food prioritisation. These results suggest that displacement might compound existing barriers and potentially increase vulnerability to micronutrient deficiencies, and highlight the need for policies that integrate cultural, economic, organisational, and psychosocial dimensions of micronutrient access.

Finally, the barriers participants encountered to getting informed about micronutrients can be interpreted through the lens of (digital) health literacy, which encompasses individuals’ ability to access, understand, appraise, and use health information to make informed decisions [46]. Participants’ accounts suggest that barriers were not primarily related to a lack of available information about micronutrients but rather to difficulties navigating an increasingly complex information landscape. Information overload, conflicting messages, and fragmented or poorly communicated information may undermine individuals’ capacity to identify, process, and use information about micronutrients, while uncertainty about the reliability of sources highlights the importance of critical appraisal skills. These challenges may be further amplified in digital environments, where nutrition information is widely available but varies substantially in quality and credibility and where misinformation can be difficult to distinguish from evidence-based advice. Together, these findings highlight the need not only to strengthen individuals’ (digital) health literacy but also to promote clearer, more accessible, and trustworthy information about micronutrients while also tackling misinformation. This may support the ability of socioeconomically disadvantaged groups to navigate complex nutrition information, make informed dietary choices, and ultimately achieve micronutrient adequacy.

### 4.1. Strengths and Limitations

Key strengths of this study are its cross-country design and the engagement of refugees and food assistance beneficiaries as primary respondents, groups that are under-represented in existing research. Furthermore, while earlier studies have predominantly examined factors related to the availability, accessibility, and affordability of healthy food, our theoretically informed analysis using the AAAQ framework highlights the often-overlooked role of perceived food quality. We showed that, although concerns about food quality (e.g., processing, freshness, premature harvesting, misleading labelling) are not always directly linked to micronutrient content, participants reported these concerns can discourage them from choosing micronutrient-rich foods even when such foods are physically or financially accessible. The study also advances understanding of how organisational food environments might affect access to and consumption of micronutrient-rich foods, and sheds light on the diverse strategies participants employed to cope with limited affordability and their information needs in this area. Finally, by exploring multiple dimensions relevant to micronutrient adequacy including consumption of micronutrient-rich foods, dietary diversity, consumption of fortified foods, use of food supplements, and sun exposure the study provides a broad account of participants’ experiences and perceptions, although it does not assess micronutrient intake or status.

This study also comes with limitations. It was conducted through charitable organisations in two cities, selected because of existing relationships with the research team, and used relatively small samples. Consequently, the findings should not be interpreted as representative of Italy or Poland or, indeed, of food-assistance beneficiaries and refugees more generally. Perspectives of refugees in Italy might have been incompletely captured because of linguistic and recruitment challenges resulting in two substantially shorter FGs, thereby limiting the breadth and depth of discussion. Conversely, the refugee FG in Poland included 12 and 13 participants, respectively, which might have limited opportunities for each participant to contribute. The exclusion of individuals who were not sufficiently fluent in Italian or Polish may have introduced selection bias, as language proficiency may be related to access to food, health information, and other resources. This potentially limits the transferability of findings to refugees and asylum seekers with limited proficiency in the local language. In addition, while the facilitators’ insider knowledge and experience with the target group could enhance rapport, it might also be a source of social desirability bias. Although facilitators received training intended to support neutral and inclusive discussion, training cannot remove the power imbalance created by their leadership roles within organisations that recruited or provided services to participants. Participants’ vulnerable positions within broader social, economic, and institutional contexts might therefore have influenced how they articulated their experiences. For example, participants may have been reluctant to criticize the services they received. This potential influence of social desirability should be considered when interpreting the findings, as it may have shaped the range of experiences participants expressed. The absence of formal pilot-testing of the discussion guide prior to data collection may have affected the clarity or comprehensiveness of the questions by the target groups. In addition, the infographic might have influenced which foods or issues participants subsequently discussed. Finally, the transcription and translation processes in this multilingual study will have affected the preservation of nuance, meaning, and culturally specific expressions. Despite efforts to ensure accuracy, some meanings might have been altered or lost, potentially influencing data interpretation. This risk is particularly relevant because the analysis was conducted using the English translations rather than the original-language transcripts. The study also relied on participants’ reported practices, perceptions, and recollections of clinical advice or test results; it did not assess dietary intake or micronutrient status and therefore cannot determine participants’ micronutrient adequacy or prevalence of deficiency.

### 4.2. Implications for Research, Policy, and Practice

The present findings suggest the potential value of targeted, context-appropriate, and multi-level interventions to improve access to and consumption of micronutrient-rich foods among socioeconomically disadvantaged populations in comparable European settings. Participants identified rising food prices as a major constraint; interventions that mitigate their effects on access to micronutrient-rich foods therefore warrant consideration. For example, food subsidies are a promising strategy for increasing the intake of micronutrient-rich foods such as fruits and vegetables [47]. Reviewing eligibility criteria for social welfare and adjusting welfare benefits to reflect changes in food prices might also improve economic access to micronutrient-rich foods. In parallel, appropriate regulatory oversight and clear communication concerning food labelling, fortification, and food supplements might enhance consumer trust and support more informed and deliberate use of available options to meet micronutrient needs. Finally, providing equitable access to health-care systems, including clearer pathways for clinically indicated assessment of suspected deficiencies, would support their identification and management, particularly among refugees.

At the community-level, initiatives that strengthen local food systems and social networks could improve access to micronutrient-rich foods. Providing opportunities for food growing, such as community gardens or allotments, might enable residents to (re-)establish food production practices that have a positive impact on fruit and vegetable intake [48], while also fostering community engagement. In addition, food-sharing programmes might help build social ties, facilitate knowledge exchange, and reduce barriers related to food skills and cultural adaptation (for refugees), for example, by promoting the preparation and consumption of culturally appropriate nutrient-dense meals. Finally, where they are affordable, digitally accessible, and available to the populations concerned, online grocery shopping and community-based delivery services might further support access to micronutrient-rich food by addressing time and mobility constraints.

In organisational settings (i.e., food assistance programmes, refugee accommodation systems, and workplaces), establishing procurement policies that prioritise micronutrient-rich foods might increase their availability and selection or consumption; previous research has associated healthy food-procurement policies with positive dietary and health outcomes [49]. Furthermore, improving food storage and preparation infrastructure, revising restrictive food policies, and including culturally appropriate food options could facilitate regular consumption of nutritious meals in workplaces and refugee shelters [13].

Finally, education, communication, and clinical guidance can also help consumers make informed decisions about micronutrient-rich foods and appropriate food supplement use. Nutrition education interventions in food assistance settings have been found to improve knowledge, cooking skills, intake of fruits and vegetables, and food security status [50]. Communication emphasising convenient and accessible food sources of micronutrients, such as frozen or canned versions of micronutrient-rich foods, might broaden awareness of available options, where these products are locally accessible and affordable. For example, consumption of frozen fruits and vegetables has been associated with higher overall intake of fruits and vegetables and greater intake of vitamin C, potassium, calcium, and magnesium [51]. In addition, communication about budget-friendly recipes and practical cooking tips might help improve the acceptability of micronutrient-rich foods while also supporting food preparation practices that preserve nutrient content. For refugees and food assistance beneficiaries, education and communication could be delivered through sources and intermediaries trusted by the populations concerned, including formal institutions, health professionals, and social workers, and adapted to different literacy levels and language needs. Finally, providing training to health professionals on how to communicate effectively about micronutrients might help counter misinformation and support consumers in making informed choices about food and food supplement use.

Findings of this pilot study point beyond the need for better food assistance or nutrition education alone. Considered alongside our recent review of national and EU-level policy responses to micronutrient deficiencies [52], these findings show how access to micronutrient-rich diets is shaped by decisions across health, social protection, migration, agriculture, education, and the wider food system. The policy review identified fragmented responses, inconsistent implementation, and policy silos that limit integration between these areas. It also found that relevant policy instruments do not consistently address micronutrient outcomes. For example, the food-assistance initiatives examined lacked explicit frameworks for evaluating micronutrient outcomes, while front-of-pack nutrition-labelling schemes focused predominantly on energy, macronutrients, sugar, and salt and did not incorporate micronutrient density.

Food assistance and social protection policies, in particular, might continue to operate primarily according to a food-sufficiency model, with success focused on the quantity of food provided and the avoidance of hunger rather than on whether the food supports nutritionally adequate diets across the life course. This wider policy gap is also reflected in nutrition labelling, public food provision, and food-assistance programmes, which do not consistently account for or evaluate micronutrient outcomes and often lack the tools needed to do so [52].

Addressing these shortcomings requires a shift from a food-sufficiency model to a nutritional-adequacy model. Micronutrient adequacy should be made an explicit objective within nutrition, food security, social-protection, migration, public-procurement, and agricultural policies. Programmes should be assessed not only by the calories or volume of food provided, but by whether they improve equitable access to diverse, micronutrient-rich diets that support health across the life course.

Minimum nutritional standards for publicly funded food provision, including food assistance, reception centres, schools, hospitals and other institutional settings, should address nutrient density, dietary variety, cultural acceptability, and the needs of groups at increased risk. These requirements should be incorporated into procurement specifications, funding agreements, and programme evaluation.

National surveillance should assess both dietary intake and micronutrient status, with findings disaggregated by socioeconomic position and food-security or vulnerability status alongside age and sex. Because population averages can conceal substantial inequalities, surveillance should be linked to clear mechanisms for reviewing the findings and determining appropriate responses, including changes to food assistance, social protection, public procurement, fortification, supplementation, clinically appropriate deficiency assessment, and access to professional advice.

Clear accountability is also needed through a named authority or formal coordination mechanism with responsibility for action across food, health, social-protection, migration and education systems, supported by shared objectives, indicators, resources, budgets, and reporting requirements. In parallel, policies outside the health sector should be assessed for their likely effects on access to micronutrient-rich foods and micronutrient intake, since decisions affecting, for example, food prices, agricultural production, retail environments, welfare provision, migrant accommodation, and charitable food provision can all improve or undermine micronutrient adequacy, directly and indirectly. Embedding such assessment within policy development would help ensure that commitments to healthy eating for all people living in the EU result in diets that nourish people rather than merely provide enough food, while also reducing avoidable health inequalities and their longer-term social and economic consequences.

## 5. Conclusions

This pilot study highlights the complex and interacting factors that may constrain access to and consumption of micronutrient-rich foods among food assistance beneficiaries and refugees in Italy and Poland. Participants described barriers operating across personal, interpersonal, organisational, contextual, and structural levels, including financial constraints, limited availability and accessibility of micronutrient-rich foods, concerns about food quality and acceptability, time and mobility constraints, social and psychosocial challenges, and difficulties navigating information about micronutrients. These barriers may be mutually reinforcing, particularly where limited resources restrict the ability to respond to preferences for fresh, minimally processed, and culturally appropriate versions of micronutrient-rich foods.

The findings also highlight important contextual differences between settings and the need to avoid one-size-fits-all approaches. However, differences in the sociodemographic composition of the samples mean that variation between settings cannot be attributed solely to differences in local food, welfare, information, or organisational environments. For refugees in particular, displacement may compound existing socioeconomic barriers through restricted access to financial resources, culturally appropriate foods, cooking facilities, healthcare, and social support. Strengthening access to micronutrient-rich diets therefore requires responses that address both the structural conditions shaping food access and the specific circumstances of populations experiencing socioeconomic disadvantage and displacement.

Importantly, this study explored perceived barriers and experiences rather than directly assessing dietary intake or micronutrient status. The findings therefore cannot establish the prevalence of micronutrient inadequacy or deficiency among participants. Nevertheless, they identify multiple pathways through which inequitable access to diverse, micronutrient-rich diets may increase vulnerability to poor micronutrient status and highlight areas for further investigation. Future research should combine qualitative approaches with dietary and biomarker assessment to determine how these barriers translate into actual micronutrient inadequacy and deficiency.

Overall, the findings support a shift from a focus on food sufficiency towards nutritional adequacy and equity. Improving micronutrient adequacy among socioeconomically disadvantaged populations will require coordinated action across food assistance, social protection, healthcare, migration, education, and food-system policies. Such efforts should improve not only the quantity of food available, but also provide equitable access to affordable and acceptable micronutrient-rich diets.

## Figures and Tables

**Figure 1:**
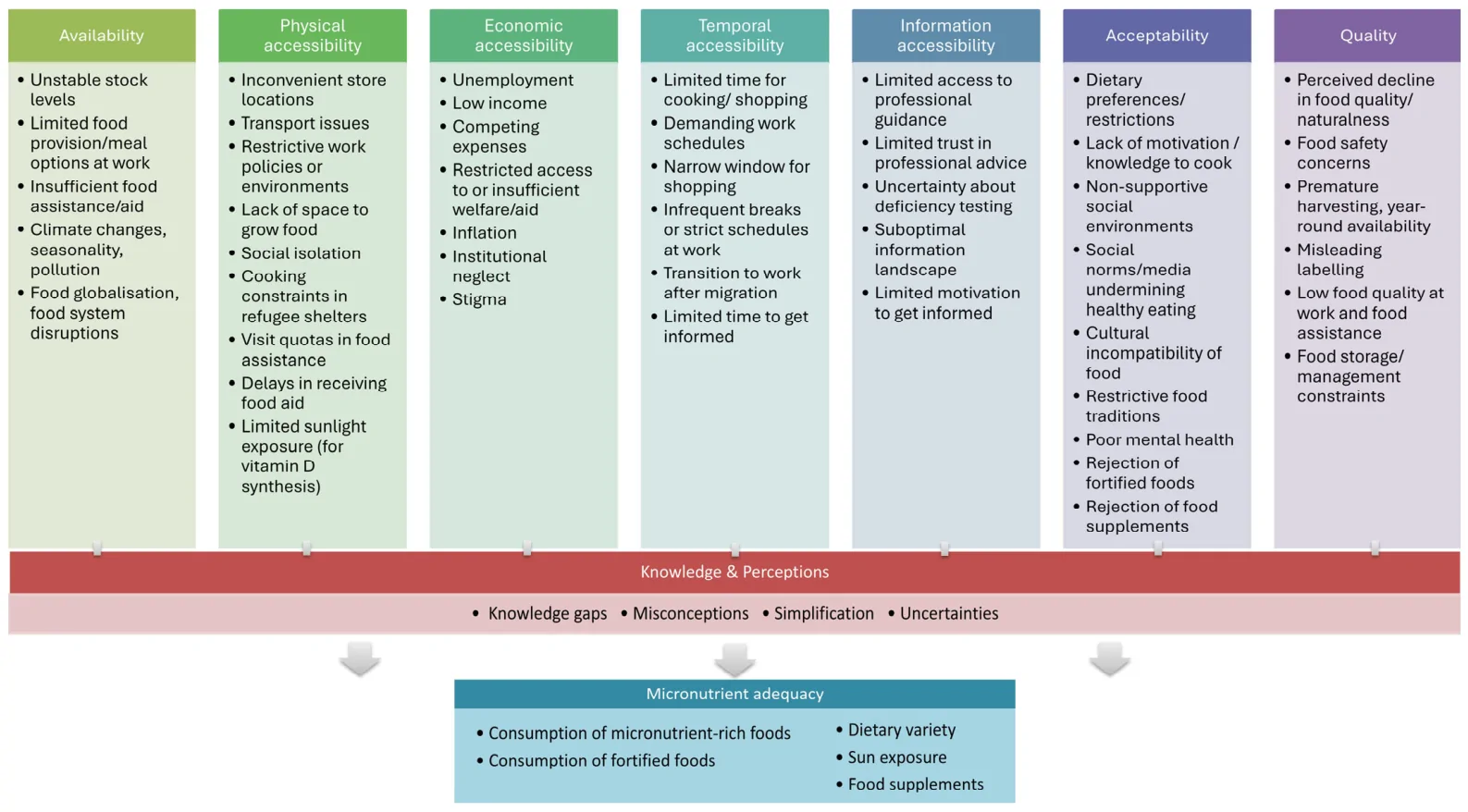
Perceived barriers to accessing and consuming micronutrient-rich foods among food assistance beneficiaries and refugees.

## Data Availability

The raw data supporting the conclusions of this article will be made available by the authors on request. The thematic framework, including themes, subthemes, codes and selected illustrative quotations, is provided in the Supplementary Materials. Full transcripts and participant-level demographic data are not publicly available because they contain sensitive information, may present a risk of participant identification, and their sharing is not covered by participant consent and ethics approval. Further inquiries may be directed to the corresponding author.

## Supplementary Materials

The following supporting information can be downloaded at: https://www.mdpi.com/article/10.3390/foods15193515/s1, Supplementary File S1: Thematic analysis; Table S1: Participant characteristics.

## Abbreviations

The following abbreviations are used in this manuscript:

AAAQ	Availability, Accessibility, Acceptability, and Quality
EU	European Union
FG	Focus group
M	Mean
Mdn	Median
