# Supplementary material for: Perceived Barriers to Micronutrient-Rich Diets Among Charitable-Service Users in Two European Settings: A Qualitative Pilot Study
Source: Foods. 2026 Oct 1;15(19):3515. doi: 10.3390/foods15193515 (PMC13665399; doi:10.3390/foods15193515)
Supplement: Supplementary file 1 [file foods-15-03515-s001.zip › Supplementary Table S1.pdf]

Table S1. Participant characteristics

	Italy (n = 26)	Poland (n = 42)	Total (N = 68)
Participant group			
Refugee / Asylum seeker	46.2%	59.5%	54.4%
Food assistance beneficiary	53.8%	40.5%	45.6%
Sex			
Male	30.8%	11.9%	19.1%
Female	69.2%	88.1%	80.9%
Age			
18 – 25	3.8%	14.3%	10.3%
26 – 35	15.4%	14.3%	14.7%
36 – 45	19.2%	23.8%	22.1%
46 – 55	11.5%	16.7%	14.7%
56 – 65	15.4%	21.4%	19.1%
66 – 75	19.2%	7.1%	11.8%
76 – 85	7.7%	-	2.9%
Prefer not to say	7.7%	2.4%	4.4%
Education			
Primary or lower education	3.8%	11.9%	8.8%
Lower secondary education	46.2%	11.9%	25.0%
Upper secondary education	23.1%	14.3%	17.6%
Post-secondary non-tertiary education	3.8%	19.0%	13.2%
Bachelor’s or equivalent	15.4%	7.1%	10.3%
Master’s or equivalent	3.8%	26.2%	17.6%
Doctorate or equivalent	-	7.1%	4.4%
Prefer not to say	3.8%	2.4%	2.9%
Employment status (multiple responses allowed)			
Temporarily unemployed	26.9%	28.6%	27.9%

Not allowed to work (e.g., due to living status, no work permit)	-	-	-
Not able to work (e.g., due to health issues)	7.7%	14.3%	11.8%
Retired	19.2%	16.7%	17.6%
Homemaker	7.7%	9.5%	8.8%
Student	7.7%	4.8%	5.9%
Employee in manual labour	15.4%	11.9%	13.2%
Employee in non-manual labour	7.7%	14.3%	11.8%
Self-employed	7.7%	7.1%	7.4%
Prefer not to say	-	2.4%	1.5%
Residence area			
Urban area (densely populated area or intermediate density area, e.g., city, town, suburbs)	92.3%	85.7%	88.2%
Rural area (thinly populated area)	-	14.3%	8.8%
Prefer not to say	7.7%	-	2.9%
Annual household income			
Less than 10,000€	65.4%	31.0%	44.1%
10,000€ - 20,000€	7.7%	19.0%	14.7%
More than 20,000€	-	14.3%	8.8%
Prefer not to say	26.9%	35.7%	32.3%
Social security benefits or disability income in the household			
Yes	26.9%	19.0%	22.1%
No	61.5%	69.0%	66.2%
Prefer not to say	11.5%	11.9%	11.8%
Household size			
1	19.2%	11.9%	14.7%
2	26.9%	26.2%	26.5%

3	26.9%	31.0%	29.4%
4	11.5%	14.3%	13.2%
5	-	2.4%	1.5%
6	7.7%	4.8%	5.9%
7	-	2.4%	1.5%
11	3.8%	-	1.5%
Prefer not to say	3.8%	7.1%	5.9%
Children in the household			
None	46.2%	45.2%	45.6%
1	30.8%	16.7%	22.1%
2	11.5%	14.3%	13.2%
3	-	7.1%	4.4%
4	-	4.8%	2.9%
Prefer not to say	11.5%	11.9%	11.8%
Migrated to Italy / Poland			
Yes	50.0%	61.9%	57.4%
No	34.6%	35.7%	35.3%
Prefer not to say	15.4%	2.4%	7.4%
Country of migration			
Bangladesh	7.7%	-	2.9%
Belarus	-	9.5%	5.9%
Nigeria	7.7%	-	2.9%
Serbia	11.5%	-	4.4%
Tunisia	3.8%	-	1.5%
Ukraine	15.4%	47.6%	35.3%
Prefer not to say	3.8%	4.8%	4.4%
Stayed in asylum seeking centre in host country			
Yes	42.3%	14.3%	25.0%

No	7.7%	42.9%	29.4%
Prefer not to say	-	4.8%	2.9%
Length of stay in asylum seeking centre			
Less than a year	-	4.8%	2.9%
1 – 2 years	11.5%	7.1%	8.8%
3 – 4 years	7.7%	-	2.9%
5 years	11.5%	-	4.4%
Prefer not to say	11.5%	2.4%	5.9%
Dietary restrictions/preferences			
None	69.2%	69.0%	69.1%
Vegetarian	-	2.4%	1.5%
Vegan	3.8%	-	1.5%
Flexitarian	-	-	-
Flexi-vegan	-	-	-
Pescatarian	-	-	-
Kosher	-	-	-
Halal	11.5%	-	4.4%
Dairy-free/ lactose-intolerant	3.8%	2.4%	2.9%
Gluten-free/ gluten-intolerant	3.8%	-	1.5%
Food allergies or other intolerances	3.8%	16.7%	11.8%
Other	7.7%	16.7%	13.2%
